# Antiviral Activity of Micafungin and Its Derivatives against SARS-CoV-2 RNA Replication

**DOI:** 10.3390/v15020452

**Published:** 2023-02-06

**Authors:** Shogo Nakajima, Hirofumi Ohashi, Daisuke Akazawa, Shiho Torii, Rigel Suzuki, Takasuke Fukuhara, Koichi Watashi

**Affiliations:** 1 Research Center for Drug and Vaccine Development, National Institute of Infectious Diseases, 1-23-1 Toyama, Shinjuku-ku, Tokyo 162-8640, Japan; 2 Department of Virology II, National Institute of Infectious Diseases, 1-23-1 Toyama, Shinjuku-ku, Tokyo 162-8640, Japan; 3 Choju Medical Institute, Fukushimura Hospital, 19-14 Yamanaka, Noyoricho, Toyohashi-shi 441-8124, Japan; 4 Laboratory of Virus Control, Research Institute for Microbial Diseases, Osaka University, Suita 565-0871, Japan; 5 Insect-Virus Interactions Unit, Department of Virology, Institut Pasteur, 75015 Paris, France; 6 Department of Microbiology and Immunology, Faculty of Medicine, Hokkaido University, N-15, W-7, Kita-ku, Sapporo 060-8638, Japan; 7 Department of Applied Biological Sciences, Tokyo University of Science, 2641 Yamazaki, Noda 278-8510, Japan

**Keywords:** severe acute respiratory syndrome coronavirus 2, micafungin, echinocandin, antiviral agent, replication

## Abstract

Echinocandin antifungal drugs, including micafungin, anidulafungin, and caspofungin, have been recently reported to exhibit antiviral effects against various viruses such as flavivirus, alphavirus, and coronavirus. In this study, we focused on micafungin and its derivatives and analyzed their antiviral activities against severe acute respiratory syndrome coronavirus 2 (SARS-CoV-2). The micafungin derivatives Mi-2 and Mi-5 showed higher antiviral activity than micafungin, with 50% maximal inhibitory concentration (IC_50_) of 5.25 and 6.51 µM, respectively (3.8 to 4.7-fold stronger than micafungin) and 50% cytotoxic concentration (CC_50_) of >64 µM in VeroE6/TMPRSS2 cells. This high anti-SARS-CoV-2 activity was also conserved in human lung epithelial cell-derived Calu-3 cells. Micafungin, Mi-2, and Mi-5 were suggested to inhibit the intracellular virus replication process; additionally, these compounds were active against SARS-CoV-2 variants, including Delta (AY.122, hCoV-19/Japan/TY11-927/2021), Omicron (BA.1.18, hCoV-19/Japan/TY38-873/2021), a variant resistant to remdesivir (R10/E796G C799F), and a variant resistant to casirivimab/imdevimab antibody cocktail (E406W); thus, our results provide basic evidence for the potential use of micafungin derivatives for developing antiviral agents.

## 1. Introduction

Coronavirus disease 2019 (COVID-19), which is caused by severe acute respiratory syndrome coronavirus 2 (SARS-CoV-2), remains a global public health emergency with more than 648 million infections and 6.6 million deaths as of December 2022 (https://covid19.who.int/, accessed on 10 December 2022). In the past 20 years, SARS-CoV, Middle East respiratory syndrome coronavirus, and SARS-CoV-2 have emerged as novel human coronaviruses, which indicates the potential threat of this viral group. There are currently several US Food and Drug Administration-approved anti-COVID-19 agents, including antiviral agents such as remdesivir (RDV), molnupiravir, and nirmatrelvir. RDV and molnupiravir are nucleoside analogues that target viral polymerases and show broad-spectrum antiviral effects; they were developed as drug candidates against viruses including ebola virus, hepatitis C viruses, and respiratory syncytia virus, before the COVID-19 pandemic [1,2,3,4,5]. Continuous development and studies on these antiviral agents enabled the rapid and successful application of these agents for treatment of COVID-19 within two years of the pandemic; thus, the identification and analysis of new classes of antiviral agents are important for not only developing a drug against the current infectious disease but also for developing a rapid response for managing new emerging infectious diseases, which should be stressed given the probable outbreaks of emerging viral infections in the future.

Micafungin (MCFG), anidulafungin, and caspofungin, belonging to the echinocandin class of antifungal drugs, are inhibitors of fungal cell wall synthesis. Fungal cell walls are composed of β-(1,3)-glucan, β-(1,4)-glucan, β-(1,6)-glucan, α-glucan, chitin, mannan, and various glycoproteins, the quantity and relative content of which vary depending on fungal species [6,7]. Echinocandins inhibit cell wall biosynthesis via noncompetitive inhibition of the β-(1,3)-glucan synthase complex [6,7,8], which is absent in mammalian cells, thereby providing a drug target with a favorable safety profile. The echinocandins are semi-synthetic lipopeptides originating from natural compounds; the original compounds of MCFG, anidulafungin, and caspofungin are FR901379 (WF11899A) [9] from *Coleophoma empetri*, echinocandin B from *Aspergillus nidulans* var. *echinulatus*, and pneumocandin B0 from *Glarea lozoyensis*, respectively [6]. Recently, echinocandin antifungal drugs have been reported to exert antiviral effects on several viruses [7,10,11,12,13,14]; MCFG and anidulafungin have been reported to inhibit SARS-CoV-2 infection [15,16]. In the present study, we focused on MCFG and its derivatives (see Figure S1) and analyzed their antiviral activities against SARS-CoV-2 and its variants. Our data present the antiviral potential of MCFG and its derivatives. The MCFG derivatives Mi-2 and Mi-5 showed higher antiviral activity than that of MCFG and may be used to develop antiviral agents.

## 2. Materials and Methods

### 2.1. Compounds

MCFG, anidulafungin, and caspofungin were obtained from Selleck Biotech (Tokyo, Japan). The MCFG derivative Mi-4 (ASP9726) was chemically synthesized as previously reported [17] and patented [18], and other derivatives (Mi-1, Mi-2, Mi-3, Mi-5, and Mi-6) were similarly synthesized as described previously [18,19,20,21]. RDV and cepharanthine (CEP) were purchased from Chemscene (Monmouth Junction, NJ, USA) and Sigma-Aldrich (St. Louis, MO, USA), respectively.

### 2.2. Cell Culture

VeroE6/TMPRSS2 cells (transmembrane serine protease 2,-overexpressing VeroE6 cells [22]) were cultured in Dulbecco’s modified Eagle’s medium (Fujifilm Wako Pure Chemical, Osaka, Japan) supplemented with 10% fetal bovine serum (FBS; Sigma-Aldrich), 100 units/mL penicillin, 100 µg/mL streptomycin, 10 mM HEPES (pH 7.4), and 1 mg/mL G418 (Nacalai Tesque, Kyoto, Japan) at 37 °C in 5% CO_2_. During the infection assay, G418 was removed and 10% FBS in the medium was replaced with 2% FBS. Calu-3 cells (human-derived lung epithelial cell line) were cultured in Eagle’s minimum essential medium (Fujifilm Wako Pure Chemical), supplemented with 10% FBS, 100 units/mL penicillin, 100 μg/mL streptomycin, 10 mM HEPES (pH 7.4), and 1% GlutaMAX^TM^ supplement (Thermo Fisher Scientific, Waltham, MA, USA).

### 2.3. Infection Assay

SARS-CoV-2 was handled at biosafety level 3. We used the SARS-CoV-2 Wuhan strain (2019-hCoV/Japan/TY/WK-521/2020, GISAID ID: EPI_ISL_408667), Delta variant AY.122 (hCoV-19/Japan/TY11-927/2021, GISAID ID: EPI_ISL_2158617), Omicron variant BA.1.18 (hCoV-19/Japan/TY38-873/2021, GISAID ID: EPI_ISL_7418017), an E406W variant that is resistant to casirivimab/imdevimab (REGN-COV2) antibody cocktail (the complete sequence was shown in Table S1) [23], and RDV-escape mutant R10/E796G C799F (GenBank ID: LC742929) [23,24]. VeroE6/TMPRSS2 cells were inoculated with the viruses at a multiplicity of infection (MOI) of 0.003 for 1 h and unbound viruses were removed by washing, as previously described [25,26]. Cells were cultured for an additional 24 h and the extracellular viral RNA was quantified via real-time reverse transcription–polymerase chain reaction (RT-PCR) analysis. Compounds were added both during the virus inoculation (1 h) and after washing the inoculum (24 h), and 10 µM RDV was used as a positive control in these assays (see Figures S2A and S3A–D). In this experimental condition, nearly 100% population of cells was SARS-CoV-2 positive at 24 h post-inoculation [26,27].

**Figure 1:**
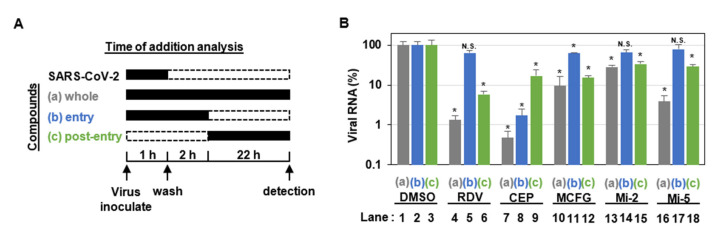
MCFG and its derivatives inhibited the post-entry process of SARS-CoV-2. (**A**) Schematic representation of the time-of-addition analysis. Compounds were added at three different times: (**a**) whole: throughout the assay for 25 h, (**b**) entry: for the initial 3 h to evaluate the effect on the viral entry process, and (**c**) post-entry: for the last 22 h to evaluate the effect on viral replication/re-infection. (**B**) Extracellular viral RNA concentration of the cells treated as shown in (**A**) was quantified via real-time RT-PCR. The cells were treated with either 5 µM RDV, 3 µM CEP, 50 µM MCFG, 8 µM Mi-2, or 8 µM Mi-5 under the three experimental conditions (**a**–**c**), as shown in (**A**). These data were from hexaplicate experiments in DMSO and triplicate experiments in samples are presented as average values with error bars indicating standard deviation. * *p* < 0.05 vs. DMSO; N.S., not significant; with Mann–Whitney U test. RDV, remdesivir; CEP, cepharanthine; MCFG, micafungin; and DMSO, dimethyl sulfoxide.

The time-of-addition analysis was performed by adding compounds at three different time points to VeroE6/TMPRSS2 cells that were inoculated with SARS-CoV-2 Wuhan strain at an MOI of 0.1 (see Figure 1A): (a, whole life cycle, gray) present during the 1 h virus inoculation and maintained throughout the 24 h infection period; (b, entry, blue) treated during the 1 h virus inoculation and for an additional 2 h and then removed; (c, post-entry, green) added at 2 h after virus inoculation and treated for the remaining 22 h until harvest. Remdesivir and cepharanthine were used as positive controls for a replication and an entry inhibitor, respectively, as reported [28,29].

The Calu-3 cell infection assay was performed by inoculating the Wuhan strain for 3 h and incubating for an additional 21 h after which the extracellular viral RNA concentration was measured and 10 µM RDV was used as a positive control in the assay (see Figure S2C).

### 2.4. Quantification of Viral RNA

Viral RNA in the culture supernatant was extracted using a MagMAX Viral/Pathogen II Nucleic Acid Isolation kit (Thermo Fisher Scientific) and quantified via real-time RT-PCR analysis using a one-step quantitative RT-PCR kit (Thunderbird Probe One-step qRT-PCR kit; Toyobo, Osaka, Japan) using the SARS-CoV-2-specific primers (Forward: 5′-ACAGGTACGTTAATAGTTAATAGCGT-3′, Reverse: 5′-ATATTGCAGCAGTACGCACACA-3′) and probe (5′-FAM-ACACTAGCCATCCTTACTGCGCTTCG-TAMRA-3′) [30]. The quantification of viral RNA was calculated by ∆∆Ct methods [31].

### 2.5. Cell Viability

Cell viability was measured via a cytotoxicity assay using cell-counting kit-8 (Dojindo laboratories, Kumamoto, Japan) according to the manufacturer’s instructions (see Figure S2B,D). This examination evaluates the cellular dehydrogenase activities by treating tetrazolium salt, WST-8, which is reduced under dehydrogenase to produce a yellow formazan dye. VeroE6/TMPRSS2 and Calu-3 cells cultured for 24 h with the tested compounds were evaluated by this assay.

### 2.6. Calculation of IC_50_, IC_90_, IC_99_, and CC_50_

Inhibitory concentrations of 50, 90, and 99% maximum as well as 50% maximal cytotoxic concentration (IC_50_, IC_90_, IC_99_, and CC_50_) of each compound were determined from a regression line [Y = AX + B] of two values that sandwich 50, 90, or 99% inhibition at compound concentration (X) and inhibition value (Y). X (IC_50_, IC_90_, IC_99_, and CC_50_ values) was calculated when Y in the regression line was substituted with 50, 90, or 99.

### 2.7. Statistics

Statistical analyses were performed using GraphPad Prism 9 software, and significance was determined using a non-parametric test (* *p* < 0.05, ** *p* < 0.01; N.S., not significant).

## 3. Results

### 3.1. MCFG and Its Derivatives Show Antiviral Activity against SARS-CoV-2 Infections

We used MCFG, anidulafungin, and caspofungin as approved echinocandin antifungal agents (see Figure S1) to evaluate anti-SARS-CoV-2 activity in a cell culture infection model [29]. Figure S2A,B show the viral RNA levels and cell viabilities, respectively, upon treatment with different concentrations of compounds. The IC_50_, IC_90,_ IC_99_, and CC_50_ were calculated and are shown in Figure S2. MCFG and anidulafungin, among the three approved echinocandin antifungal agents, reduced the viral RNA levels in a dose-dependent manner (IC_50_ = 26.1 and 7.09 µM, respectively), while caspofungin did not show any antiviral effect up to a concentration of 64 µM (see Figure S2A). The cell viability assay showed a dose-dependent cytotoxicity of anidulafungin (CC_50_ = 24.6 µM) (see Figure S2B). Since anidulafungin had a narrow window between anti-SARS-CoV-2 activity and cytotoxicity, we focused on MCFG and synthesized derivatives. We synthesized six MCFG derivatives, Mi-1, Mi-2, Mi-3, Mi-4, Mi-5, and Mi-6 by changing the side chains of MCFG (see Materials and Methods, and Figure S1). In the SARS-CoV-2 infection assay, Mi-1, Mi-2, Mi-3, Mi-4, and Mi-5 reduced the viral RNA concentration without apparent cytotoxicity, while Mi-6 had no effect on viral RNA levels up to 64 µM (see Figure S2A,B). Among them, Mi-2 and Mi-5 showed lower IC_50_s than that of MCFG (IC_50_ = 5.25 and 6.51 µM, respectively; 3.8 to 4.7-fold lower than MCFG), but no remarkable reduction in cell viability (see Figure S2A,B); further, in Calu-3 cells, Mi-2 and Mi-5 showed stronger antiviral activity than MCFG (see Figure S2C, IC_50_ = 55.3, 10.1, and 5.71 µM for MCFG, Mi-2, and Mi-5, respectively), but with a detectable toxicity for Mi-5 (see Figure S2D, CC_50_ = 48.5 µM) (see Table 1); thus, we produced new derivatives of MCFG that have higher antiviral potencies.

### 3.2. MCFG and Its Derivatives Inhibit SARS-CoV-2 Replication

To determine the stages in the SARS-CoV-2 life cycle inhibited by MCFG, Mi-2, and Mi-5, we performed a time-of-addition assay in which antiviral activities were evaluated after treatment with the compounds at three different times (see Figure 1A, a–c). In this assay, RDV, a reported SARS-CoV-2 replication inhibitor [32], was used as a positive control and showed no inhibitory effect when added at the virus entry phase (see Figure 1B, lane 5), but showed remarkable antiviral activity at the post-entry phase (see Figure 1B, lane 6). We also confirmed the mode of action of CEP, a SARS-CoV-2 entry inhibitor that inhibits SARS-CoV-2 particle binding to cells [29], based on considerable inhibition of the virus entry phase (see Figure 1B, lane 8) as well as a lower viral activity at post-entry phase. This is likely to be obtained by inhibition of multiple rounds of viral re-infection (see Figure 1B, lane 9) [29]. In this assay, Mi-2 and Mi-5 clearly reduced viral RNA levels when added during the whole life cycle and the post-entry phase, but exhibited no inhibitory effect on the virus entry phase, similar to the effects of RDV (see Figure 1B, lanes 10–18). These data suggest that MCFG, Mi-2, and Mi-5 target virus replication, rather than the process for viral entry.

### 3.3. Antiviral Activity of MCFG and Its Derivatives against SARS-CoV-2 Variants

We further examined whether MCFG, Mi-2, and Mi-5 were effective against SARS-CoV-2 variants (see Figure S3). MCFG, Mi-2, and Mi-5 were effective in reducing viral RNA concentrations of Delta (IC_50_ = 11.8, <2, and <2 µM, respectively), Omicron (IC_50_ = 55.9, 13.0, and 7.56 µM, respectively), E406W (IC_50_ = 21.7, <2, and 4.48 µM, respectively), and R10/E796G C799F (IC_50_ = 31.3, 9.67, and 7.22 µM, respectively) (see Table 2). These data indicated that MCFG, Mi-2, and Mi-5 have wide antiviral effects on multiple SARS-CoV-2 variants, and Mi-2 and Mi-5 showed stronger antiviral activities than MCFG against all SARS-CoV-2 variants examined.

## 4. Discussion

Echinocandin antifungal drugs have been reported to show antiviral activities against multiple viruses. Kim et al. reported that MCFG inhibits Enterovirus 71 (EV71) infection in the first study describing the antiviral activity of an echinocandin compound [10]. This study suggested that MCFG targets virus replication and reported an estimated IC_50_ of 5–8 µM to a luciferase carrying EV71 replicon in Vero cells and weaker antiviral activity against coxsackievirus group B type 3-infected Hela cells and human rhinovirus-infected H1HeLa cells [10]. Recently, Ho et al. reported that MCFG inhibits the infection of Chikungunya virus (CHIKV) using U2OS or BHK-21 cells [11], Dengue virus serotype 2 (DENV-2) in Vero cells [12], and Zika virus (ZIKV) in Vero cells [14] (IC_50_ = 20.63, 10.23, and 7.35 µM, respectively). They also reported that anidulafungin inhibits DENV-2 [12] and ZIKV infections [14] (IC_50_ = 3.24 and 2.08 µM, respectively) and that caspofungin inhibits DENV-2 [12] and ZIKV [14] infections in the parallel infection assays (IC_50_ = 20.78 and 75.75 µM, respectively). MCFG and anidulafungin have also been reported to inhibit SARS-CoV-2 infection in Vero cells with IC_50_ = 5.5–12.9 µM [15] and 4.64 µM [16], respectively, consistent with the anti-SARS-CoV-2 activities shown in the present study (see Figure S2A and Table 1). Regarding the target of antiviral action of these echinocandin drugs, MCFG inhibits the replication of CHIKV [11] and the entry of DENV-2 [12], while anidulafungin inhibits ZIKV entry [14], which is mainly demonstrated in time-of-addition assays; also, MCFG was reported to inhibit the helicase enzymatic activity of DENV-2 in vitro (IC_50_ = 4.98 µM) [13], in addition to its anti-DENV entry activity [12], speculating that MCFG may potentially have multiple targets for antiviral activity. In a structural computer modeling of SARS-CoV-2 proteins, MCFG has been predicted to interact with 3CLpro [33,34] and was shown to inhibit 3CLpro enzymatic activity in vitro (IC_50_ = 47.63 µM) [33]. It should be clarified whether 3CLpro is an actual target for MCFG, Mi-2, and Mi-5 in the future. Additionally, anidulafungin inhibits angiotensin converting enzyme 2-spike protein interaction in SARS-CoV-2 pseudovirus entry assay [35]; thus, MCFG and the related echinocandins may have multiple targets as broad-spectrum antiviral agents. Given the possible reduced efficacy of antiviral activity against emerging SARS-CoV-2 variants, development of additional anti-SARS-CoV-2 drugs with new mode of action is demanded. Identification of the target molecule of MCFG and its derivatives would be needed in the future.

**Figure 2:**
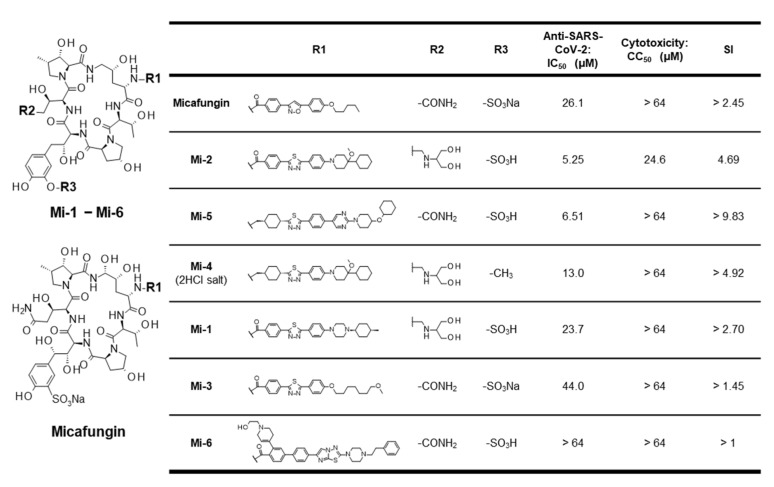
Structure–activity relationship of micafungin derivatives with anti-SARS-CoV-2 activity, cytotoxicity, and selectivity index (SI = CC_50_/IC_50_) observed in VeroE6/TMPRSS2 cells. The base structure (**left**) and the moieties at R1, R2, and R3 (**right**) are shown.

In this study, we summarized the structure–activity relationship of the MCFG derivatives in Figure 2. Comparison of MCFG (IC_50_ = 26.1 µM), Mi-5 (IC_50_ = 6.51 µM), and Mi-6 (IC_50_ > 64 µM), which share the same basic skeleton, R2, and R3 with different structure in R1, indicated the importance of the R1 structure for anti-SARS-CoV-2 activity. Since Mi-2, Mi-5, Mi-4, and Mi-1 had higher anti-SARS-CoV-2 activities than that of MCFG, replacement of the R1 moiety of MCFG with structures containing two six-membered rings (cyclohexane, piperidine, or piperazine) might increase its activity. By comparing Mi-2 and Mi-4 that have identical R2 and similar R1, the SO_3_H group in R3 seems preferable for high anti-SARS-CoV-2 activity. Further derivative analysis based on the aforementioned structure–activity relationship is expected to yield more potent antiviral compounds.

The pharmacokinetics of MCFG for human adults indicate that the C_max_ and half-life are, reportedly, 7.2 mg/L (5.67 µM) and 11–17 h upon administration at 100 mg daily, respectively; 100 mg/d is the approved dose for anti-fungal treatment [7]. The exposure concentration of MCFG in serum in treated adults is lower than the anti-SARS-CoV-2 IC_50_ calculated in this study, but that in the human lung has not been well determined. In addition, the pharmacokinetics profiles of the MCFG derivatives are needed to predict the antiviral potency in an in vivo setting. Further development of MCFG derivatives may be useful not only for developing anti-SARS-CoV-2 drugs but also as broad-spectrum antiviral drugs, which are helpful for developing a rapid response to probable new virus pandemics in the future.

## Figures and Tables

**Table 1:** The IC_50_ and CC_50_ of Micafungin and its derivatives against a SARS-CoV-2 Wuhan strain.

Compound	VeroE6/TMPRSS2 Cells		Calu-3 Cells	
	SARS-CoV-2 RNA	Cell Viability	SARS-CoV-2 RNA	Cell Viability
	IC_50_ (µM)	CC_50_ (µM)	IC_50_ (µM)	CC_50_ (µM)
Micafungin	26.1	>64	55.3	>64
Anidulafungin	7.09	24.6	-	-
Caspofungin	>64	>64	-	-
Mi-1	23.7	>64	-	-
Mi-2	5.25	>64	10.1	>64
Mi-3	44.0	>64	-	-
Mi-4	13.0	>64	-	-
Mi-5	6.51	>64	5.71	48.5
Mi-6	>64	>64	-	-
Remdesivir *	1.58	>64	-	-
Cepharanthine *	0.35	25.1	-	-

* Data from Ohashi et al. [29] essentially in the same experimental condition.

**Table 2:** The IC_50_ against SARS-CoV-2 variants.

Compound	SARS-CoV-2 RNA	SARS-CoV-2 RNA	SARS-CoV-2 RNA	SARS-CoV-2 RNA
	Delta	Omicron	E406W	R10/E796G C799F
	IC_50_ (µM)	IC_50_ (µM)	IC_50_ (µM)	IC_50_ (µM)
Micafungin	11.8	55.9	21.7	31.3
Mi-2	<2	13.0	<2	9.67
Mi-5	<2	7.56	4.48	7.22

## Data Availability

The data presented in this study are available on request from the corresponding author.

## Supplementary Materials

The following supporting information can be downloaded at: https://www.mdpi.com/article/10.3390/v15020452/s1, Figure S1: Chemical structures of micafungin, caspofungin, anidulafungin, and the micafungin derivatives; Figure S2: Anti-SARS-CoV-2 activities of micafungin and its derivatives; Figure S3: Activities of micafungin and its derivatives against SARS-CoV-2 variants; Table S1: The complete sequence of SARS-CoV-2 E406W variant.
