# Supplementary material for: Antiviral Activity of Micafungin and Its Derivatives against SARS-CoV-2 RNA Replication
Source: Viruses. 2023 Feb 6;15(2):452. doi: 10.3390/v15020452 (PMC9958940; doi:10.3390/v15020452)
Supplement: Supplementary file 1 [file viruses-15-00452-s001.zip › viruses-2138775-supplementary.pdf]

Supplementary Material

Micafungin

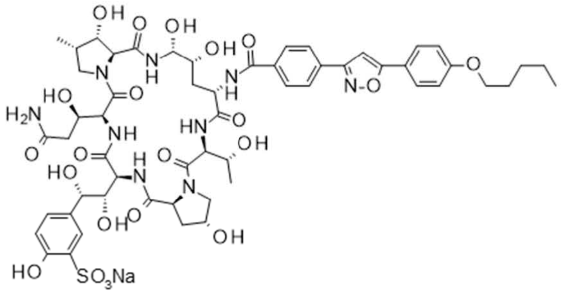

Caspofungin

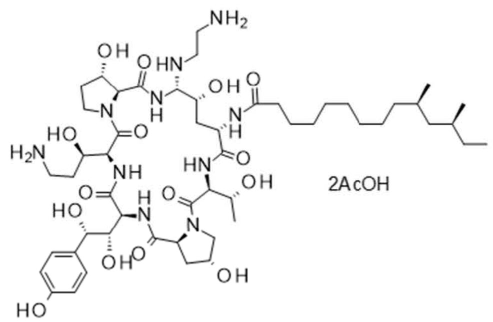

Anidulafungin

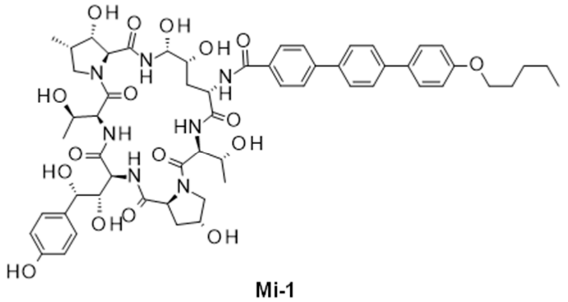

Mi-1

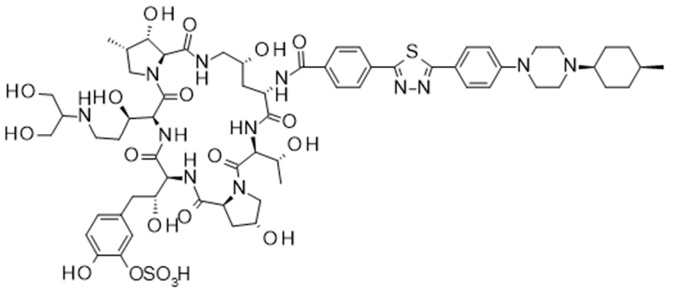

Mi-2

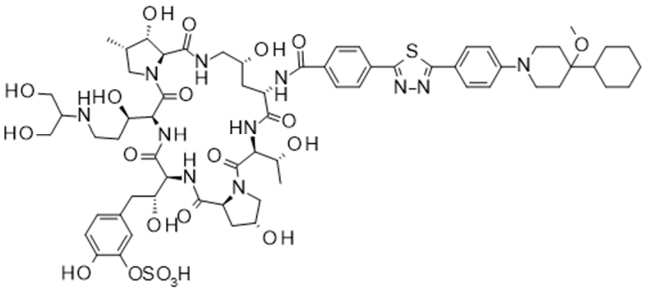

Mi-3

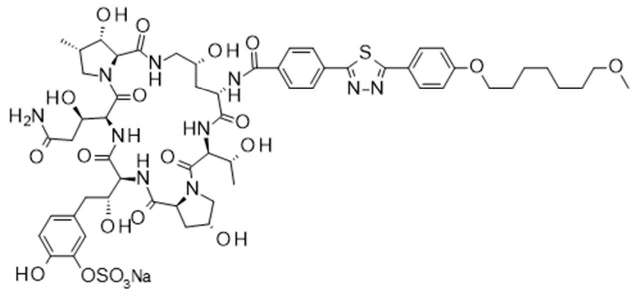

Mi-4

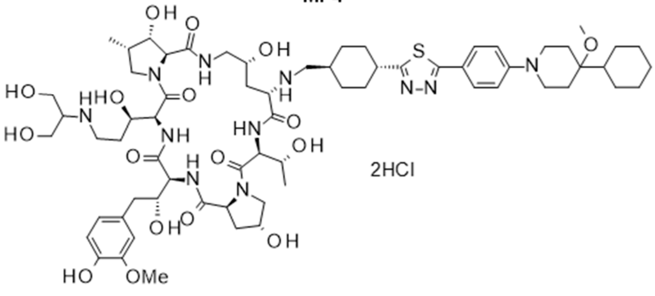

Mi-5

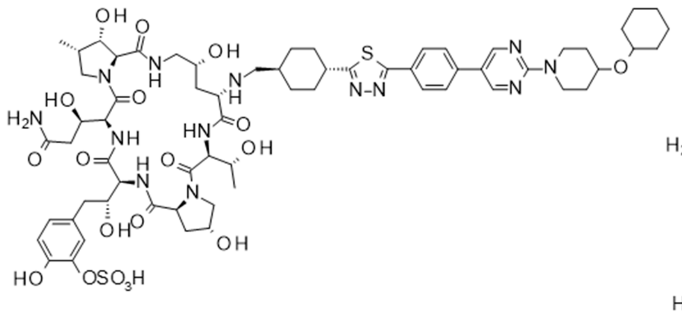

Mi-6

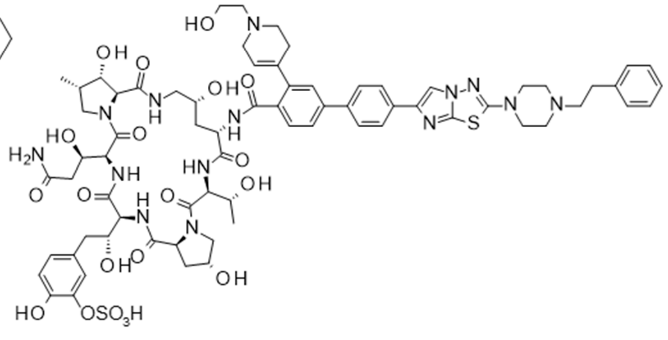

Figure S1. Chemical structures of micafungin, caspofungin, anidulafungin, and the micafungin derivatives, Mi-1, Mi-2, Mi-3, Mi-4, Mi-5, and Mi-6.

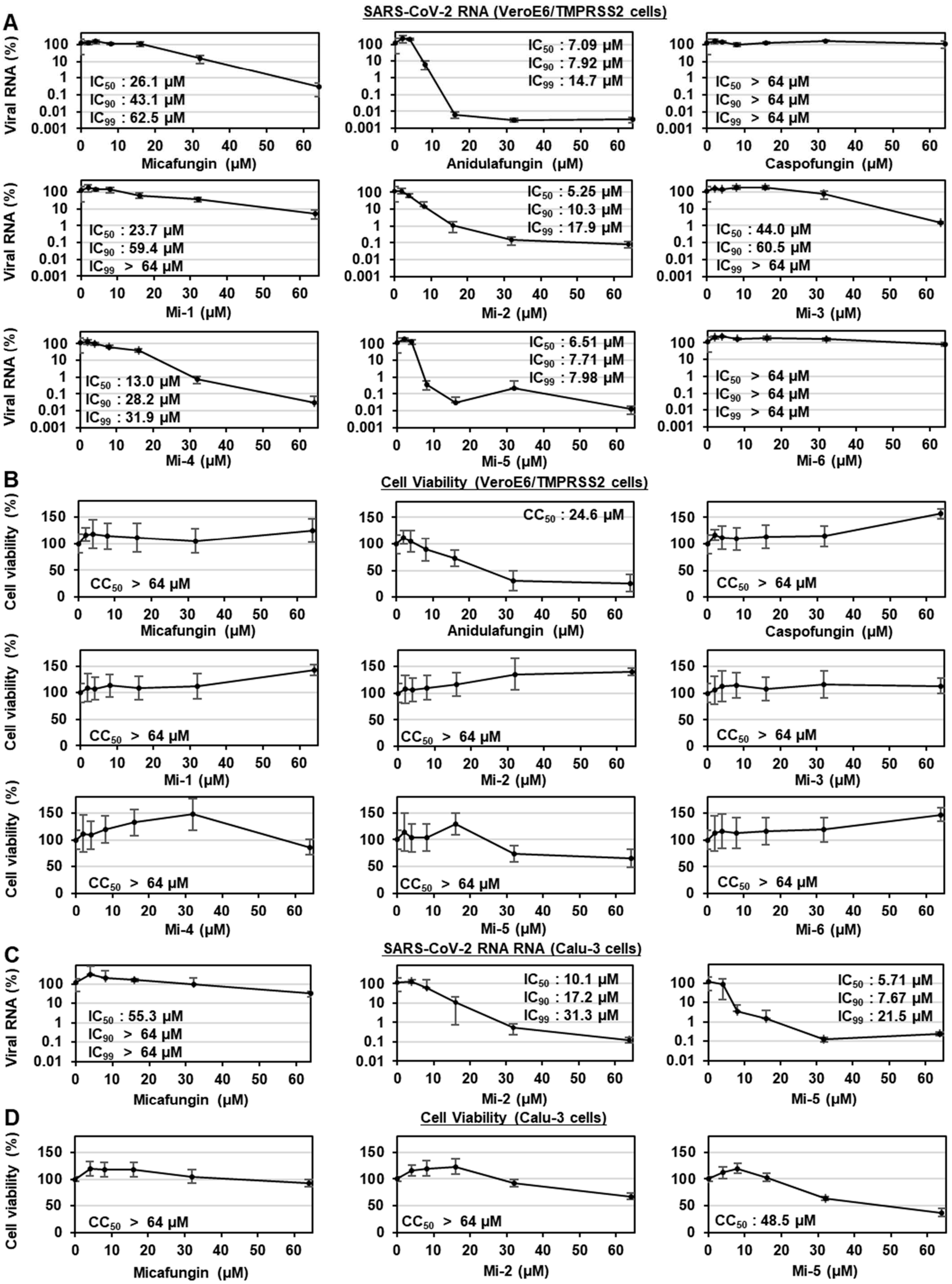
A
SARS-CoV-2 RNA (VeroE6/TMPRSS2 cells)
Viral RNA (%)
IC50 : 26.1 µM
IC90 : 43.1 µM
IC99 : 62.5 µM
Micafungin (µM)
IC50 : 7.09 µM
IC90 : 7.92 µM
IC99 : 14.7 µM
Anidulafungin (µM)
IC50 > 64 µM
IC90 > 64 µM
IC99 > 64 µM
Caspofungin (µM)
IC50 : 23.7 µM
IC90 : 59.4 µM
IC99 > 64 µM
Mi-1 (µM)
IC50 : 5.25 µM
IC90 : 10.3 µM
IC99 : 17.9 µM
Mi-2 (µM)
IC50 : 44.0 µM
IC90 : 60.5 µM
IC99 > 64 µM
Mi-3 (µM)
IC50 : 13.0 µM
IC90 : 28.2 µM
IC99 : 31.9 µM
Mi-4 (µM)
IC50 : 6.51 µM
IC90 : 7.71 µM
IC99 : 7.98 µM
Mi-5 (µM)
IC50 > 64 µM
IC90 > 64 µM
IC99 > 64 µM
Mi-6 (µM)
B
Cell Viability (VeroE6/TMPRSS2 cells)
Cell viability (%)
CC50 > 64 µM
Micafungin (µM)
CC50 : 24.6 µM
Anidulafungin (µM)
CC50 > 64 µM
Caspofungin (µM)
CC50 > 64 µM
Mi-1 (µM)
CC50 > 64 µM
Mi-2 (µM)
CC50 > 64 µM
Mi-3 (µM)
CC50 > 64 µM
Mi-4 (µM)
CC50 > 64 µM
Mi-5 (µM)
CC50 > 64 µM
Mi-6 (µM)
C
SARS-CoV-2 RNA RNA (Calu-3 cells)
Viral RNA (%)
IC50 : 55.3 µM
IC90 > 64 µM
IC99 > 64 µM
Micafungin (µM)
IC50 : 10.1 µM
IC90 : 17.2 µM
IC99 : 31.3 µM
Mi-2 (µM)
IC50 : 5.71 µM
IC90 : 7.67 µM
IC99 : 21.5 µM
Mi-5 (µM)
D
Cell Viability (Calu-3 cells)
Cell viability (%)
CC50 > 64 µM
Micafungin (µM)
CC50 > 64 µM
Mi-2 (µM)
CC50 : 48.5 µM
Mi-5 (µM)

Figure S2. Anti-SARS-CoV-2 activities of micafungin and its derivatives. (A) VeroE6/TMPRSS2 cells were treated with 2, 4, 8, 16, 32, and 64 µM of each compound during 1-hour virus inoculation and for 24 h after inoculation. Extracellular SARS-CoV-2 RNA concentration at 24 h post-inoculation was quantified via real time RT-PCR. Relative amounts of SARS-CoV-2 RNA (log in Y-axis) against the compound concentration (X-axis) are shown as graphs. (B) VeroE6/TMPRSS2 cell viabilities after treatment for 24 h were determined using the cytotoxicity assay explained in the Materials and Methods. (C, D) A human-derived lung epithelial cell line, Calu-3 cells, was treated with the indicated compound at concentrations of 2, 4, 8, 16, 32, and 64 µM to quantify extracellular SARS-CoV-2 RNA (C) and cell viability (D), as shown in (A) and (B). The calculated IC50, IC90, IC99 and CC50 for each compound are also indicated. The black line indicates the average data of three independent experiments. SARS-CoV-2, severe acute respiratory syndrome coronavirus 2; IC50, half maximal inhibitory concentration; IC90, 90% maximal inhibitory concentration; IC99, 99% maximal inhibitory concentration; and CC50, half maximal cytotoxic concentration. All data are shown with error bars indicating S.D.

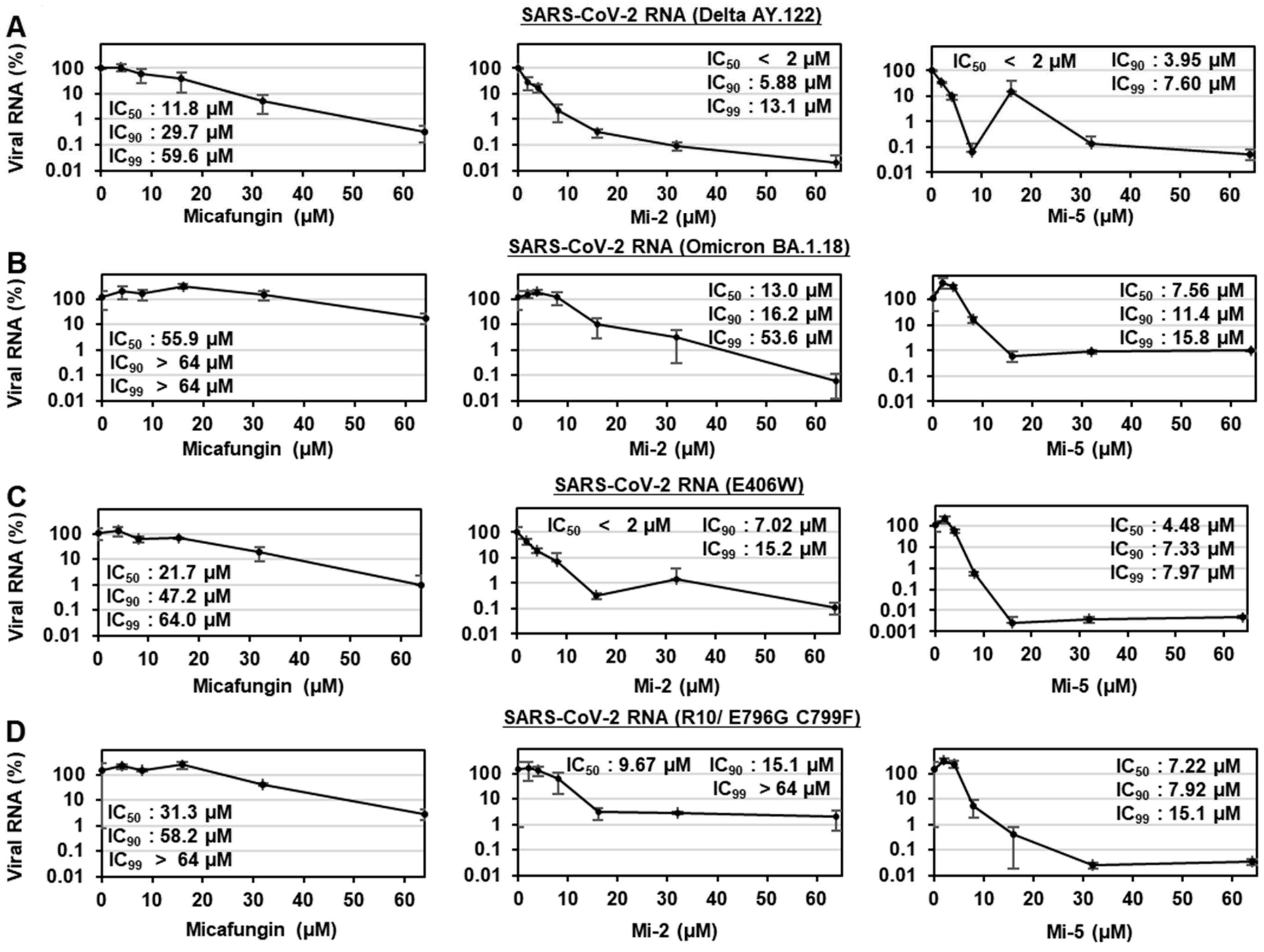

Figure S3. Activities of micafungin and its derivatives against SARS-CoV-2 variants. Extracellular SARS-CoV-2 RNA level in VeroE6/TMPRSS2 cells infected with either Delta (A), Omicron (B), a variant resistant

to the casirivimab/imdevimab antibody cocktail (C), or a variant resistant to remdesivir (D), was determined upon treatment with micafungin (4, 8, 16, 32, and 64 μM), Mi-2, and Mi-5 (2, 4, 8, 16, 32, and 64 μM). These data were from three independent experiments. The black line indicates the average data of three independent experiments. All data are shown with error bars indicating S.D.

Table. S1. The complete sequence of SARS-CoV-2 E406W variant.

1 attaaaggtt tataccttcc caggtaacaa accaaccaac tttcgatctc ttgtagatct
61 gttctctaaa cgaactttaa aatctgtgtg gctgtcactc ggctgcatgc ttagtgcact
121 cacgcagtat aattaataac taattactgt cgttgacagg acacgagtaa ctcgtctatc
181 ttctgcaggc tgcttacggt ttcgtccgtg ttgcagccga tcatcagcac atctaggttt
241 cgtccgggtg tgaccgaaag gtaagatgga gagccttgtc cctggtttca acgagaaaac
301 acacgtccaa ctcagtttgc ctgttttaca ggttcgcgac gtgctcgtac gtggctttgg
361 agactccgtg gaggaggtct tatcagaggc acgtcaacat cttaaagatg gcacttgtgg
421 cttagtagaa gttgaaaaag gcgttttgcc tcaacttgaa cagccctatg tgttcatcaa
481 acgttcggat gctcgaactg cacctcatgg tcatgttatg gttgagctgg tagcagaact
541 cgaaggcatt cagtacggtc gtagtggtga gacacttggt gtccttgtcc ctcatgtggg
601 cgaaatacca gtggcttacc gcaaggttct tcttcgtaag aacggtaata aaggagctgg
661 tggccatagt tacggcgccg atctaaagtc atttgactta ggcgacgagc ttggcactga
721 tccttatgaa gattttcaag aaaactggaa cactaaacat agcagtggtg ttacccgtga
781 actcatgcgt gagcttaacg gaggggcata cactcgctat gtcgataaca acttctgtgg
841 ccctgatggc taccctcttg agtgcattaa agaccttcta gcacgtgctg gtaaagcttc
901 atgcactttg tccgaacaac tggactttat tgacactaag aggggtgtat actgctgccg
961 tgaacatgag catgaaattg cttggtacac ggaacgttct gaaaagagct atgaattgca
1021 gacacctttt gaaattaaat tggcaaagaa atttgacacc ttcaatgggg aatgtccaaa
1081 ttttgtattt cccttaaatt ccataatcaa gactattcaa ccaagggttg aaaagaaaaa
1141 gcttgatggc tttatgggta gaattcgatc tgtctatcca gttgcgtcac caaatgaatg
1201 caaccaaatg tgcctttcaa ctctcatgaa gtgtgatcat tgtggtgaaa cttcatggca
1261 gacgggcgat tttgttaaag ccacttgcga attttgtggc actgagaatt tgactaaaga
1321 aggtgccact acttgtggtt acttacccca aaatgctgtt gttaaaattt attgtccagc
1381 atgtcacaat tcagaagtag gacctgagca tagtcttgcc gaataccata atgaatctgg
1441 cttgaaaacc attcttcgta agggtggtcg cactattgcc tttggaggct gtgtgttctc
1501 ttatgttggt tgccataaca agtgtgccta ttgggttcca cgtgctagcg ctaacatagg
1561 ttgtaaccat acaggtgttg ttggagaagg ttccgaaggt cttaatgaca accttcttga
1621 aatactccaa aaagagaaag tcaacatcaa tattgttggt gactttaaac ttaatgaaga
1681 gatcgccatt attttggcat ctttttctgc ttccacaagt gcttttgtgg aaactgtgaa
1741 aggtttggat tataaagcat tcaaacaaat tgttgaatcc tgtggtaatt ttaaagttac
1801 aaaaggaaaa gctaaaaaag gtgcctggaa tattggtgaa cagaaatcaa tactgagtcc
1861 tctttatgca tttgcatcag aggctgctcg tgttgtacga tcaattttct cccgcactct
1921 tgaaactgct caaaattctg tgcgtgtttt acagaaggcc gctataacaa tactagatgg

1981 aatttcacag tattcactga gactcattga tgctatgatg ttcacatctg atttggctac
2041 taacaatcta gttgtaatgg cctacattac aggtggtgtt gttcagttga cttcgcagtg
2101 gctaactaac atctttggca ctgtttatga aaaactcaaa cccgtccttg attggcttga
2161 agagaagttt aaggaaggtg tagagtttct tagagacggt tgggaaattg ttaaatttat
2221 ctcaacctgt gcttgtgaaa ttgtcggtgg acaaattgtc acctgtgcaa aggaaattaa
2281 ggagagtgtt cagacattct ttaagcttgt aaataaattt ttggctttgt gtgctgactc
2341 tatcattatt ggtggagcta aacttaaagc cttgaattta ggtgaaacat ttgtcacgca
2401 ctcaaaggga ttgtacagaa agtgtgttaa atccagagaa gaaactggcc tactcatgcc
2461 tctaaaagcc ccaaaagaaa ttatcttctt agagggagaa acacttccca cagaagtgtt
2521 aacagaggaa gttgtcttga aaactggtga tttacaacca ttagaacaac ctactagtga
2581 agctgttgaa gctccattgg ttggtacacc agtttgtatt aacgggctta tgttgctcga
2641 aatcaaagac acagaaaagt attgtgccct tgcacctaat atgatggtaa caaacaatac
2701 cttcacactc aaaggcggtg caccaacaaa ggttactttt ggtgatgaca ctgtgataga
2761 agtgcaaggt tacaagagtg tgaatatcac ttttgaactt gatgaaagga ttgataaagt
2821 acttaatgag aagtgctctg cctatacagt tgaactcggt acagaagtaa atgagttcgc
2881 ctgtgttgtg gcagatgctg tcataaaaac tttgcaacca gtatctgaat tacttacacc
2941 actgggcatt gatttagatg agtggagtat ggctacatac tacttatttg atgagtctgg
3001 tgagtttaaa ttggcttcac atatgtattg ttctttctac cctccagatg aggatgaaga
3061 agaaggtgat tgtgaagaag aagagtttga gccatcaact caatatgagt atggtactga
3121 agatgattac caaggtaaac ctttggaatt tggtgccact tctgctgctc ttcaacctga
3181 agaagagcaa gaagaagatt ggttagatga tgatagtcaa caaactgttg gtcaacaaga
3241 cggcagtgag gacaatcaga caactactat tcaaacaatt gttgaggttc aacctcaatt
3301 agagatggaa cttacaccag ttgttcagac tattgaagtg aatagtttta gtggttattt
3361 aaaacttact gacaatgtat acattaaaaa tgcagacatt gtggaagaag ctaaaaaggt
3421 aaaaccaaca gtggttgtta atgcagccaa tgtttacctt aaacatggag gaggtgttgc
3481 aggagcctta aataaggcta ctaacaatgc catgcaagtt gaatctgatg attacatagc
3541 tactaatgga ccacttaaag tgggtggtag ttgtgtttta agcggacaca atcttgctaa
3601 acactgtctt catgttgtcg gcccaaatgt taacaaaggt gaagacattc aacttcttaa
3661 gagtgcttat gaaaatttta atcagcacga agttctactt gcaccattat tatcagctgg
3721 tatttttggt gctgacccta tacattcttt aagagtttgt gtagatactg ttcgcacaaa
3781 tgtctactta gctgtctttg ataaaaatct ctatgacaaa cttgtttcaa gctttttgga
3841 aatgaagagt gaaaagcaag ttgaacaaaa gatcgctgag attcctaaag aggaagttaa
3901 gccatttata actgaaagta aaccttcagt tgaacagaga aaacaagatg ataagaaaat
3961 caaagcttgt gttgaagaag ttacaacaac tctggaagaa actaagttcc tcacagaaaa
4021 cttgttactt tatattgaca ttaatggcaa tcttcatcca gattctgcca ctcttgttag
4081 tgacattgac atcactttct taaagaaaga tgctccatat atagtgggtg atgttgttca
4141 agagggtgtt ttaactgctg tggttatacc tactaaaaag gctggtggca ctactgaaat
4201 gctagcgaaa gctttgagaa aagtgccaac agacaattat ataaccactt acccgggtca
4261 gggtttaaat ggttacactg tagaggaggc aaagacagtg cttaaaaagt gtaaaagtgc
4321 cttttacatt ctaccatcta ttatctctaa tgagaagcaa gaaattcttg gaactgtttc

4381 ttggaatttg cgagaaatgc ttgcacatgc agaagaaaca cgcaaattaa tgcctgtctg
4441 tgtggaaact aaagccatag tttcaactat acagcgtaaa tataagggta ttaaaataca
4501 agagggtgtg gttgattatg gtgctagatt ttacttttac accagtaaaa caactgtagc
4561 gtcacttatc aacacactta acgatctaaa tgaaactctt gttacaatgc cacttggcta
4621 tgtaacacat ggcttaaatt tggaagaagc tgctcggtat atgagatctc tcaaagtgcc
4681 agctacagtt tctgtttctt cacctgatgc tgttacagcg tataatggtt atcttacttc
4741 ttcttctaaa acacctgaag aacattttat tgaaaccatc tcacttgctg gttcctataa
4801 agattggtcc tattctggac aatctacaca actaggtata gaatttctta agagaggtga
4861 taaaagtgta tattacacta gtaatcctac cacattccac ctagatggtg aagttatcac
4921 ctttgacaat cttaagacac ttctttcttt gagagaagtg aggactatta aggtgtttac
4981 aacagtagac aacattaacc tccacacgca agttgtggac atgtcaatga catatggaca
5041 acagtttggt ccaacttatt tggatggagc tgatgttact aaaataaaac ctcataattc
5101 acatgaaggt aaaacatttt atgttttacc taatgatgac actctacgtg ttgaggcttt
5161 tgagtactac cacacaactg atcctagttt tctgggtagg tacatgtcag cattaaatca
5221 cactaaaaag tggaaatacc cacaagttaa tggtttaact tctattaaat gggcagataa
5281 caactgttat cttgccactg cattgttaac actccaacaa atagagttga agtttaatcc
5341 acctgctcta caagatgctt attacagagc aagggctggt gaagctgcta acttttgtgc
5401 acttatctta gcctactgta ataagacagt aggtgagtta ggtgatgtta gagaaacaat
5461 gagttacttg tttcaacatg ccaatttaga ttcttgcaaa agagtcttga acgtggtgtg
5521 taaaacttgt ggacaacagc agacaaccct taagggtgta gaagctgtta tgtacatggg
5581 cacactttct tatgaacaat ttaagaaagg tgttcagata ccttgtacgt gtggtaaaca
5641 agctacaaaa tatctagtac aacaggagtc accttttgtt atgatgtcag caccacctgc
5701 tcagtatgaa cttaagcatg gtacatttac ttgtgctagt gagtacactg gtaattacca
5761 gtgtggtcac tataaacata taacttctaa agaaactttg tattgcatag acggtgcttt
5821 acttacaaag tcctcagaat acaaaggtcc tattacggat gttttctaca aagaaaacag
5881 ttacacaaca accataaaac cagttactta taaattggat ggtgttgttt gtacagaaat
5941 tgaccctaag ttggacaatt attataagaa agacaattct tatttcacag agcaaccaat
6001 tgatcttgta ccaaaccaac catatccaaa cgcaagcttc gataatttta agtttgtatg
6061 tgataatatc aaatttgctg atgatttaaa ccagttaact ggttataaga aacctgcttc
6121 aagagagctt aaagttacat ttttccctga cttaaatggt gatgtggtgg ctattgatta
6181 taaacactac acaccctctt ttaagaaagg agctaaattg ttacataaac ctattgtttg
6241 gcatgttaac aatgcaacta ataaagccac gtataaacca aatacctggt gtatacgttg
6301 tctttggagc acaaaaccag ttgaaacatc aaattcgttt gatgtactga agtcagagga
6361 cgcgcaggga atggataatc ttgcctgcga agatctaaaa ccagtctctg aagaagtagt
6421 ggaaaatcct accatacaga aagacgttct tgagtgtaat gtgaaaacta ccgaagttgt
6481 aggagacatt atacttaaac cagcaaataa tagtttaaaa attacagaag aggttggcca
6541 cacagatcta atggctgctt atgtagacaa ttctagtctt actattaaga aacctaatga
6601 attatctaga gtattaggtt tgaaaaccct tgctactcat ggtttagctg ctgttaatag
6661 tgtcccttgg gatactatag ctaattatgc taagcctttt cttaacaaag ttgttagtac
6721 aactactaac atagttacac ggtgtttaaa ccgtgtttgt actaattata tgccttattt

6781 ctttacttta ttgctacaat tgtgtacttt tactagaagt acaaattcta gaattaaagc
6841 atctatgccg actactatag caaagaatac tgttaagagt gtcggtaaat tttgtctaga
6901 ggcttcattt aattatttga agtcacctaa tttttctaaa ctgataaata ttataatttg
6961 gtttttacta ttaagtgttt gcctaggttc tttaatctac tcaaccgctg ctttaggtgt
7021 tttaatgtct aatttaggca tgccttctta ctgtactggt tacagagaag gctatttgaa
7081 ctctactaat gtcactattg caacctactg tactggttct ataccttgta gtgtttgtct
7141 tagtggttta gattctttag acacctatcc ttctttagaa actatacaaa ttaccatttc
7201 atcttttaaa tgggatttaa ctgcttttgg cttagttgca gagtggtttt tggcatatat
7261 tcttttcact aggtttttct atgtacttgg attggctgca atcatgcaat tgtttttcag
7321 ctattttgca gtacatttta ttagtaattc ttggcttatg tggttaataa ttaatcttgt
7381 acaaatggcc ccgatttcag ctatggttag aatgtacatc ttctttgcat cattttatta
7441 tgtatggaaa agttatgtgc atgttgtaga cggttgtaat tcatcaactt gtatgatgtg
7501 ttacaaacgt aatagagcaa caagagtcga atgtacaact attgttaatg gtgttagaag
7561 gtccttttat gtctatgcta atggaggtaa aggcttttgc aaactacaca attggaattg
7621 tgttaattgt gatacattct gtgctggtag tacatttatt agtgatgaag ttgcgagaga
7681 cttgtcacta cagtttaaaa gaccaataaa tcctactgac cagtcttctt acatcgttga
7741 tagtgttaca gtgaagaatg gttccatcca tctttacttt gataaagctg gtcaaaagac
7801 ttatgaaaga cattctctct ctcattttgt taacttagac aacctgagag ctaataacac
7861 taaaggttca ttgcctatta atgttatagt tttgatggt aaatcaaaat gtgaagaatc
7921 atctgcaaaa tcagcgtctg tttactacag tcagcttatg tgtcaaccta tactgttact
7981 agatcaggca ttagtgtctg atgttggtga tagtgcggaa gttgcagtta aaatgtttga
8041 tgcttacgtt aatacgtttt catcaacttt taacgtacca atggaaaaac tcaaaacact
8101 agttgcaact gcagaagctg aacttgcaaa gaatgtgtcc ttagacaatg tcttatctac
8161 ttttatttca gcagctcggc aagggtttgt tgattcagat gtagaaacta aagatgttgt
8221 tgaatgtctt aaattgtcac atcaatctga catagaagtt actggcgata gttgtaataa
8281 ctatatgctc acctataaca aagttgaaaa catgacaccc cgtgaccttg gtgcttgtat
8341 tgactgtagt gcgcgtcata ttaatgcgca ggtagcaaaa agtcacaaca ttgctttgat
8401 atggaacgtt aaagatttca tgtcattgtc tgaacaacta cgaaaacaaa tacgtagtgc
8461 tgctaaaaag aataacttac cttttaagtt gacatgtgca actactagac aagttgttaa
8521 tgttgtaaca acaaagatag cacttaaggg tggtaaaatt gttaataatt ggttgaagca
8581 gttaattaaa gttacacttg tgttcctttt tgttgctgct attttctatt taataacacc
8641 tgttcatgtc atgtctaaac atactgactt ttcaagtgaa atcataggat acaaggctat
8701 tgatggtggt gtcactcgtg acatagcatc tacagatact tgttttgcta acaaacatgc
8761 tgattttgac acatggttta gtcagcgtgg tggtagttat actaatgaca aagcttgccc
8821 attgattgct gcagtcataa caagagaagt gggttttgtc gtgcctggtt tgcctggcac
8881 gatattacgc acaactaatg gtgacttttt gcatttctta cctagagttt ttagtgcagt
8941 tggtaacatc tgttacacac catcaaaact tatagagtac actgactttg caacatcagc
9001 ttgtgttttg gctgctgaat gtacaatttt taaagatgct tctggtaagc cagtaccata
9061 ttgttatgat accaatgtac tagaaggttc tgttgcttat gaaagtttac gccctgacac
9121 acgttatgtg ctcatggatg gctctattat tcaatttcct aacacctacc ttgaaggttc

9181 tgttagagtg gtaacaactt ttgattctga gtactgtagg cacggcactt gtgaaagatc
9241 agaagctggt gtttgtgtat ctactagtgg tagatgggta cttaacaatg attattacag
9301 atctttacca ggagttttct gtggtgtaga tgctgtaaat ttacttacta atatgtttac
9361 accactaatt caacctattg gtgctttgga catatcagca tctatagtag ctggtggtat
9421 tgtagctatc gtagtaacat gccttgccta ctattttatg aggtttagaa gagcttttgg
9481 tgaatacagt catgtagttg cctttaatac tttactattc cttatgtcat tcactgtact
9541 ctgtttaaca ccagtttact cattcttacc tggtgtttat tctgttattt acttgtactt
9601 gacattttat cttactaatg atgtttcttt tttagcacat attcagtgga tggttatgtt
9661 cacaccttta gtacctttct ggataacaat tgcttatatc atttgtattt ccacaaagca
9721 tttctattgg ttctttagta attacctaaa gagacgtgta gtctttaatg gtgtttcctt
9781 tagtactttt gaagaagctg cgctgtgcac ctttttgtta aataaagaaa tgtatctaaa
9841 gttgcgtagt gatgtgctat tacctcttac gcaatataat agatacttag ctctttataa
9901 taagtacaag tattttagtg gagcaatgga tacaactagc tacagagaag ctgcttgttg
9961 tcatctcgca aaggctctca atgacttcag taactcaggt tctgatgttc tttaccaacc
10021 accacaaacc tctatcacct cagctgtttt gcagagtggt tttagaaaaa tggcattccc
10081 atctggtaaa gttgagggtt gtatggtaca agtaacttgt ggtacaacta cacttaacgg
10141 tctttggctt gatgacgtag tttactgtcc aagacatgtg atctgcacct ctgaagacat
10201 gcttaaccct aattatgaag atttactcat tcgtaagtct aatcataatt tcttggtaca
10261 ggctggtaat gttcaactca gggttattgg acattctatg caaaattgtg tacttaagct
10321 taaggttgat acagccaatc ctaagacacc taagtataag tttgttcgca ttcaaccagg
10381 acagactttt tcagtgttag cttgttacaa tggttcacca tctggtgttt accaatgtgc
10441 tatgaggccc aatttcacta ttaagggttc attccttaat ggttcatgtg gtagtgttgg
10501 ttttaacata gattatgact gtgtctcttt ttgttacatg caccatatgg aattaccaac
10561 tggagttcat gctggcacag acttagaagg taacttttat ggaccttttg ttgacaggca
10621 aacagcacaa gcagctggta cggacacaac tattacagtt aatgttttag cttggttgta
10681 cgctgctgtt ataaatggag acaggtggtt tctcaatcga tttaccacaa ctcttaatga
10741 ctttaacctt gtggctatga agtacaatta tgaacctcta acacaagacc atgttgacat
10801 actaggacct ctttctgctc aaactggaat tgccgtttta gatatgtgtg cttcattaaa
10861 agaattactg caaaatggta tgaatggacg taccatattg ggtagtgctt tattagaaga
10921 tgaatttaca ccttttgatg ttgttagaca atgctcaggt gttactttcc aaagtgcagt
10981 gaaaagaaca atcaagggta cacaccactg gttgttactc acaattttga cttcactttt
11041 agttttagtc cagagtactc aatggtcttt gttctttttt ttgtatgaaa atgccttttt
11101 accttttgct atgggtatta ttgctatgtc tgcttttgca atgatgtttg tcaaacataa
11161 gcatgcattt ctctgtttgt tttgttacc ttctcttgcc actgtagctt attttaatat
11221 ggtctatatg cctgctagtt gggtgatgcg tattatgaca tggttggata tggttgatac
11281 tagtttgtct ggttttaagc taaaagactg tgttatgtat gcatcagctg tagtgttact
11341 aatccttatg acagcaagaa ctgtgtatga tgatggtgct aggagagtgt ggacacttat
11401 gaatgtcttg acactcgttt ataaagttta ttatggtaat gctttagatc aagccatttc
11461 catgtgggct cttataatct ctgttacttc taactactca ggtgtagtta caactgtcat
11521 gtttttggcc agaggtattg tttttatgtg tgttgagtat tgccctattt tcttcataac

11581 tggtaataca cttcagtgta taatgctagt ttattgtttc ttaggctatt tttgtacttg
11641 ttactttggc ctcttttgtt tactcaaccg ctactttaga ctgactcttg gtgtttatga
11701 ttacttagtt tctacacagg agtttagata tatgaattca cagggactac tcccacccaa
11761 gaatagcata gatgccttca aactcaacat taaattgttg ggtgttggtg gcaaaccttg
11821 tatcaaagta gccactgtac agtctaaaat gtcagatgta aagtgcacat cagtagtctt
11881 actctcagtt ttgcaacaac tcagagtaga atcatcatct aaattgtggg ctcaatgtgt
11941 ccagttacac aatgacattc tcttagctaa agatactact gaagcctttg aaaaaatggt
12001 ttcactactt tctgttttgc tttccatgca gggtgctgta gacataaaca agctttgtga
12061 agaaatgctg gacaacaggg caaccttaca agctatagcc tcagagttta gttcccttcc
12121 atcatatgca gcttttgcta ctgctcaaga agcttatgag caggctgttg ctaatggtga
12181 ttctgaagtt gttcttaaaa agttgaagaa gtctttgaat gtggctaaat ctgaatttga
12241 ccgtgatgca gccatgcaac gtaagttgga aaagatggct gatcaagcta tgacccaaat
12301 gtataaacag gctagatctg aggacaagag ggcaaaagtt actagtgcta tgcagacaat
12361 gcttttcact atgcttagaa agttggataa tgatgcactc aacaacatta tcaacaatgc
12421 aagagatggt tgtgttccct tgaacataat acctcttaca acagcagcca aactaatggt
12481 tgtcatacca gactataaca catataaaaa tacgtgtgat ggtacaacat ttacttatgc
12541 atcagcattg tgggaaatcc aacaggttgt agatgcagat agtaaaattg ttcaacttag
12601 tgaaattagt atggacaatt cacctaattt agcatggcct cttattgtaa cagctttaag
12661 ggccaattct gctgtcaaat tacagAATAA TGAGCTTAGT CCTGTTGCAC TACGACAGAT
12721 GTCTTGTGCT GCCGGTACTA CACAAACTGC TTGCACTGAT GACAATGCGT TAGCTTACTA
12781 CAACACAACA AAGGGAGGTA GGTTTGTACT TGCACTGTTA TCCGATTTAC AGGATTTGAA
12841 ATGGGCTAGA TTCCCTAAGA GTGATGGAAC TGGTACTATC TATACAGAAC TGGAACCACC
12901 TTGTAGGTTT GTTACAGACA CACCTAAAGG TCCTAAAGTG AAGTATTTAT ACTTTATTAA
12961 AGGATTAAAC AACCTAAATA GAGGTATGGT ACTTGGTAGT TTAGCTGCCA CAGTACGTCT
13021 ACAAgctggt aatgcaacag aagtgcctgc caattcaact gtattatctt tctgtgcttt
13081 tgctgtagat gctgctaaag cttacaaaga ttatctagct agtgggggac aaccaatcac
13141 taattgtgtt aagatgttgt gtacacacac tggtactggt caggcaataa cagttacacc
13201 ggaagccaat atggatcaag aatcctttgg tggtgcatcg tgttgtctgt actgccgttg
13261 ccacatagat catccaaatc ctaaaggatt ttgtgactta aaaggtaagt atgtacaaat
13321 acctacaact tgtgctaatg accctgtggg ttttacactt aaaaacacag tctgtaccgt
13381 ctgcggtatg tggaaaggtt atggctgtag ttgtgatcaa ctccgcgaac ccatgcttca
13441 gtcagctgat gcacaatcgt ttttaaacgg gtttgcggtg taagtgcagc ccgtcttaca
13501 ccgtgcggca caggcactag tactgatgtc gtatacaggg cttttgacat ctacaatgat
13561 aaagtagctg gttttgctaa attcctaaaa actaattgtt gtcgcttcca agaaaaggac
13621 gaagatgaca atttaattga ttcttacttt gtagttaaga gacacacttt ctctaactac
13681 caacatgaag aaacaattta taatttactt aaggattgtc cagctgttgc taaacatgac
13741 ttctttaagt ttagaataga cggtgacatg gtaccacata tatcacgtca acgtcttact
13801 aaatacacaa tggcagacct cgtctatgct ttaaggcatt ttgatgaagg taattgtgac
13861 acattaaaag aaatacttgt cacatacaat tgttgtgatg atgattattt caataaaaag
13921 gactggtatg attttgtaga aaacccagat atattacgcg tatacgccaa cttaggtgaa

13981 cgtgtacgcc aagctttgtt aaaaacagta caattctgtg atgccatgcg aaatgctggt
14041 attgttggtg tactgacatt agataatcaa gatctcaatg gtaactggta tgatttcggt
14101 gatttcatac aaaccacgcc aggtagtgga gttcctgttg tagattctta ttattcattg
14161 ttaatgccta tattaacctt gaccagggct ttaactgcag agtcacatgt tgacactgac
14221 ttaacaaagc cttacattaa gtgggatttg ttaaaatatg acttcacgga agagaggtta
14281 aaactctttg accgttattt taaatattgg gatcagacat accacccaaa ttgtgttaac
14341 tgtttggatg acagatgcat tctgcattgt gcaaacttta atgttttatt ctctacagtg
14401 ttcccaccta caagttttgg accactagtg agaaaaatat ttgttgatgg tgttccattt
14461 gtagtttcaa ctggatacca cttcagagag ctaggtgttg tacataatca ggatgtaaac
14521 ttacatagct ctagacttag ttttaaggaa ttacttgtgt atgctgctga ccctgctatg
14581 cacgctgctt ctggtaatct attactagat aaacgcacta cgtgcttttc agtagctgca
14641 cttactaaca atgttgcttt tcaaactgtc aaacccggta attttaacaa agacttctat
14701 gactttgctg tgtctaaggg tttctttaag gaaggaagtt ctgttgaatt aaaacacttc
14761 ttctttgctc aggatggtaa tgctgctatc agcgattatg actactatcg ttataatcta
14821 ccaacaatgt gtgatatcag acaactacta tttgtagttg aagttgttga taagtacttt
14881 gattgttacg atggtggctg tattaatgct aaccaagtca tcgtcaacaa cctagacaaa
14941 tcagctggtt ttccatttaa taaatggggt aaggctagac tttattatga ttcaatgagt
15001 tatgaggatc aagatgcact tttcgcatat acaaaacgta atgtcatccc tactataact
15061 caaatgaatc ttaagtatgc cattagtgca aagaatagag ctcgcaccgt agctggtgtc
15121 tctatctgta gtactatgac caatagacag tttcatcaaa aattattgaa atcaatagcc
15181 gccactagag gagctactgt agtaattgga acaagcaaat tctatggtgg ttggcacaac
15241 atgttaaaaa ctgtttatag tgatgtagaa aaccctcacc ttatgggttg ggattatcct
15301 aaatgtgata gagccatgcc taacatgctt agaattatgg cctcacttgt tcttgctcgc
15361 aaacatacaa cgtgttgtag cttgtcacac cgtttctata gattagctaa tgagtgtgct
15421 caagtattga gtgaaatggt catgtgtggc ggttcactat atgttaaacc aggtggaacc
15481 tcatcaggag atgccacaac tgcttatgct aatagtgttt ttaacatttg tcaagctgtc
15541 acggccaatg ttaatgcact tttatctact gatggtaaca aaattgccga taagtatgtc
15601 cgcaatttac aacacagact ttatgagtgt ctctatagaa atagagatgt tgacacagac
15661 tttgtgaatg agttttacgc atatttgcgt aaacatttct caatgatgat actctctgac
15721 gatgctgttg tgtgtttcaa tagcacttat gcatctcaag gtctagtggc tagcataaag
15781 aactttaagt cagttcttta ttatcaaaac aatgttttta tgtctgaagc aaaatgttgg
15841 actgagactg accttactaa aggacctcat gaattttgct ctcaacatac aatgctagtt
15901 aaacagggtg atgattatgt gtaccttcct tacccagatc catcaagaat cctaggggcc
15961 ggctgttttg tagatgatat cgtaaaaaca gatggtacac ttatgattga acggttcgtg
16021 tctttagcta tagatgctta cccacttact aaacatccta atcaggagta tgctgatgtc
16081 tttcatttgt acttacaata cataagaaag ctacatgatg agttaacagg acacatgtta
16141 gacatgtatt ctgttatgct tactaatgat aacacttcaa ggtattggga acctgagttt
16201 tatgaggcta tgtacacacc gcatacagtc ttacaggctg ttggggcttg tgttctttgc
16261 aattcacaga cttcattaag atgtggtgct tgcatacgta gaccattctt atgttgtaaa
16321 tgctgttacg accatgtcat atcaacatca cataaattag tcttgtctgt taatccgtat

16381 gtttgcaatg ctccaggttg tgatgtcaca gatgtgactc aactttactt aggaggtatg
16441 agctattatt gtaaatcaca taaaccaccc attagttttc cattgtgtgc taatggacaa
16501 gtttttggtt tatataaaaa tacatgtgtt ggtagcgata atgttactga ctttaatgca
16561 attgcaacat gtgactggac aaatgctggt gattacattt tagctaacac ctgtactgaa
16621 agactcaagc tttttgcagc agaaacgctc aaagctactg aggagacatt taaactgtct
16681 tatggtattg ctactgtacg tgaagtgctg tctgacagag aattacatct ttcatgggaa
16741 gttggtaaac ctagaccacc acttaaccga aattatgtct ttactggtta tcgtgtaact
16801 aaaaacagta aagtacaaat aggagagtac acctttgaaa aaggtgacta tggtgatgct
16861 gttgtttacc gaggtacaac aacttacaaa ttaaatgttg gtgattattt tgtgctgaca
16921 tcacatacag taatgccatt aagtgcacct acactagtgc cacaagagca ctatgttaga
16981 attactggct tatacccaac actcaatatc tcagatgagt tttctagcaa tgttgcaaat
17041 tatcaaaagg ttggtatgca aaagtattct acactccagg gaccacctgg tactggtaag
17101 agtcattttg ctattggcct agctctctac tacccttctg ctcgcatagt gtatacagct
17161 tgctctcatg ccgctgttga tgcactatgt gagaaggcat taaaatattt gcctatagat
17221 aaatgtagta gaattatacc tgcacgtgct cgtgtagagt gttttgataa attcaaagtg
17281 aattcaacat tagaacagta tgtcttttgt actgtaaatg cattgcctga gacgacagca
17341 gatatagttg tctttgatga aatttcaatg gccacaaatt atgatttgag tgttgtcaat
17401 gccagattac gtgctaagca ctatgtgtac attggcgacc ctgctcaatt acctgcacca
17461 cgcacattgc taactaaggg cacactagaa ccagaatatt tcaattcagt gtgtagactt
17521 atgaaaacta taggtccaga catgttcctc ggaacttgtc ggcgttgtcc tgctgaaatt
17581 gttgacactg tgagtgcttt ggtttatgat aataagctta aagcacataa agacaaatca
17641 gctcaatgct ttaaaatgtt ttataagggt gttatcacgc atgatgtttc atctgcaatt
17701 aacaggccac aaataggcgt ggtaagagaa ttccttacac gtaaccctgc ttggagaaaa
17761 gctgtcttta tttcacctta taattcacag aatgctgtag cctcaaagat tttgggacta
17821 ccaactcaaa ctgttgattc atcacagggc tcagaatatg actatgtcat attcactcaa
17881 accactgaaa cagctcactc ttgtaatgta aacagattta atgttgctat taccagagca
17941 aaagtaggca tactttgcat aatgtctgat agagaccttt atgacaagtt gcaatttaca
18001 agtcttgaaa ttccacgtag gaatgtggca actttacaag ctgaaaatgt aacaggactc
18061 tttaaagatt gtagtaaggt aatcactggg ttacatccta cacaggcacc tacacacctc
18121 agtgttgaca ctaaattcaa aactgaaggt ttatgtgttg acatacctgg catacctaag
18181 gacatgacct atagaagact catctctatg atgggtttta aaatgaatta tcaagttaat
18241 ggttacccta acatgtttat cacccgcgaa gaagctataa gacatgtacg tgcatggatt
18301 ggcttcgatg tcgaggggtg tcatgctact agagaagctg ttggtaccaa tttaccttta
18361 cagctaggtt tttctacagg tgttaaccta gttgctgtac ctacaggtta tgttgataca
18421 cctaataata cagatttttc cagagttagt gctaaaccac cgcctggaga tcaatttaaa
18481 cacctcatac cacttatgta caaaggactt ccttggaatg tagtgcgtat aaagattgta
18541 caaatgttaa gtgacacact taaaaatctc tctgacagag tcgtatttgt cttatgggca
18601 catggctttg agttgacatc tatgaagtat tttgtgaaaa taggacctga gcgcacctgt
18661 tgtctatgtg atagacgtgc cacatgcttt tccactgctt cagacactta tgcctgttgg
18721 catcattcta ttggatttga ttacgtctat aatccgttta tgattgatgt tcaacaatgg

18781 ggttttacag gtaacctaca aagcaaccat gatctgtatt gtcaagtcca tggtaatgca
18841 catgtagcta gttgtgatgc aatcatgact aggtgtctag ctgtccacga gtgctttgtt
18901 aagcgtgttg actggactat tgaatatcct ataattggtg atgaactgaa gattaatgcg
18961 gcttgtagaa aggttcaaca catggttgtt aaagctgcat tattagcaga caaattccca
19021 gttcttcacg acattggtaa ccctaaagct attaagtgtg tacctcaagc tgatgtagaa
19081 tggaagttct atgatgcaca gccttgtagt gacaaagctt ataaaataga agaattattc
19141 tattcttatg ccacacattc tgacaaattc acagatggtg tatgcctatt ttggaattgc
19201 aatgtcgata gatatcctgc taattccatt gtttgtagat ttgacactag agtgctatct
19261 aaccttaact tgcctggttg tgatggtggc agtttgtatg taaataaaca tgcattccac
19321 acaccagctt ttgataaaag tgcttttgtt aatttaaaac aattaccatt tttctattac
19381 tctgacagtc catgtgagtc tcatggaaaa caagtagtgt cagatataga ttatgtacca
19441 ctaaagtctg ctacgtgtat aacacgttgc aatttaggtg gtgctgtctg tagacatcat
19501 gctaatgagt acagattgta tctcgatgct tataacatga tgatctcagc tggctttagc
19561 ttgtgggttt acaaacaatt tgatacttat aacctctgga acacttttac aagacttcag
19621 agtttagaaa atgtggcttt taatgttgta aataagggac actttgatgg acaacagggt
19681 gaagtaccag tttctatcat taataacact gtttacacaa aagttgatgg tgttgatgta
19741 gaattgtttg aaaataaaac aacattacct gttaatgtag catttgagct ttgggctaag
19801 cgcaacatta aaccagtacc agaggtgaaa atactcaata atttgggtgt ggacattgct
19861 gctaatactg tgatctggga ctacaaaaga gatgctccag cacatatatc tactattggt
19921 gtttgttcta tgactgacat agccaagaaa ccaactgaaa cgatttgtgc accactcact
19981 gtcttttttg atggtagagt tgatggtcaa gtagacttat ttagaaatgc ccgtaatggt
20041 gttcttatta cagaaggtag tgttaaaggt ttacaaccat ctgtaggtcc caaacaagct
20101 agtcttaatg gagtcacatt aattggagaa gccgtaaaaa cacagttcaa ttattataag
20161 aaagttgatg gtgttgtcca acaattacct gaaacttact ttactcagag tagaaattta
20221 caagaattta aacccaggag tcaaatggaa attgatttct tagaattagc tatggatgaa
20281 ttcattgaac ggtataaatt agaaggctat gccttcgaac atatcgttta tggagatttt
20341 agtcatagtc agttaggtgg tttacatcta ctgattggac tagctaaacg ttttaaggaa
20401 tcaccttttg aattagaaga ttttattcct atggacagta cagttaaaaa ctatttcata
20461 acagatgcgc aaacaggttc atctaagtgt gtgtgttctg ttattgattt attacttgat
20521 gattttgttg aaataataaa atcccaagat ttatctgtag tttctaaggt tgtcaaagtg
20581 actattgact atacagaaat ttcatttatg ctttggtgta aagatggcca tgtagaaaca
20641 ttttacccaa aattacaatc tagtcaagcg tggcaaccgg gtgttgctat gcctaatctt
20701 tacaaaatgc aaagaatgct attagaaaag tgtgaccttc aaaattatgg tgatagtgca
20761 acattaccta aaggcataat gatgaatgtc gcaaaatata ctcaactgtg tcaatattta
20821 aacacattaa cattagctgt accctataat atgagagtta tacattttgg tgctggttct
20881 gataaaggag ttgcaccagg tacagctgtt ttaagacagt ggttgcctac gggtacgctg
20941 cttgtcgatt cagatcttaa tgactttgtc tctgatgcag attcaacttt gattggtgat
21001 tgtgcaactg tacatacagc taataaatgg gatctcatta ttagtgatat gtacgaccct
21061 aagactaaaa atgttacaaa agaaaatgac tctaaagagg gtttttcac ttacatttgt
21121 gggtttatac aacaaaagct agctcttgga ggttccgtgg ctataaagat aacagaacat

21181 tcttggaatg ctgatcttta taagctcatg ggacacttcg catggtggac agcctttgtt
21241 actaatgtga atgcgtcatc atctgaagca tttttaattg gatgtaatta tcttggcaaa
21301 ccacgcgaac aaatagatgg ttatgtcatg catgcaaatt acatattttg gaggaataca
21361 aatccaattc agttgtcttc ctattcttta tttgacatga gtaaatttcc ccttaaatta
21421 aggggtactg ctgttatgtc tttaaaagaa ggtcaaatca atgatatgat tttatctctt
21481 cttagtaaag gtagacttat aattagagaa aacaacagag ttgttatttc tagtgatgtt
21541 cttgttaaca actaaacgaa caatgtttgt ttttcttgtt ttattgccac tagtctctag
21601 tcagtgtgtt aatcttacaa ccagaactca attaccccct gcatacacta attctttcac
21661 acgtggtgtt tattaccctg acaaagtttt cagatcctca gttttacatt caactcagga
21721 cttgttctta cctttctttt ccaatgttac ttggttccat gctatacatg tctctgggac
21781 caatggtact aagaggtttg ataaccctgt cctaccattt aatgatggtg tttattttgc
21841 ttccactgag aagtctaaca taataagagg ctggattttt ggtactactt tagattcgaa
21901 gacccagtcc ctacttattg ttaataacgc tactaatgtt gttattaaag tctgtgaatt
21961 tcaattttgt aatgatccat ttttgggtgt ttattaccac aaaaacaaca aaagttggat
22021 ggaaagtgag ttcagagttt attctagtgc gaataattgc acttttgaat atgtctctca
22081 gccttttctt atggaccttg aaggaaaaca gggtaatttc aaaaatctta gggaatttgt
22141 gtttaagaat attgatggtt attttaaaat atattctaag cacacgccta ttaatttagt
22201 gcgtgatctc cctcagggtt tttcggcttt agaaccattg gtagatttgc caataggtat
22261 taacatcact aggtttcaaa ctttacttgc tttacataga agttatttga ctcctggtga
22321 ttcttcttca ggttggacag ctggtgctgc agcttattat gtgggttatc ttcaacctag
22381 gacttttcta ttaaaatata atgaaaatgg aaccattaca gatgctgtag actgtgcact
22441 tgaccctctc tcagaaacaa agtgtacgtt gaaatccttc actgtagaaa aaggaatcta
22501 tcaaacttct aactttagag tccaaccaac agaatctatt gttagatttc ctaatattac
22561 aaacttgtgc ccttttggtg aagtttttaa cgccaccaga tttgcatctg tttatgcttg
22621 gaacaggaag agaatcagca actgtgttgc tgattattct gtcctatata attccgcatc
22681 attttccact tttaagtgtt atggagtgtc tcctactaaa ttaaatgatc tctgctttac
22741 taatgtctat gcagattcat ttgtaattag aggtgatTGG gtcagacaaa tcgctccagg
22801 gcaaactgga aagattgctg attataatta taaattacca gatgatttta caggctgcgt
22861 tatagcttgg aattctaaca atcttgattc taaggttggt ggtaattata attacctgta
22921 tagattgttt aggaagtcta atctcaaacc ttttgagaga gatatttcaa ctgaaatcta
22981 tcaggccggt agcacacctt gtaatggtgt tgaaggtttt aattgttact ttcctttaca
23041 atcatatggt ttccaaccca ctaatggtgt tggttaccaa ccatacagag tagtagtact
23101 ttcttttgaa cttctacatg caccagcaac tgtttgtgga cctaaaaagt ctactaattt
23161 ggttaaaaac aaatgtgtca atttcaactt caatggttta acaggcacag gtgttcttac
23221 tgagtctaac aaaaagtttc tgcctttcca acaatttggc agagacattg ctgacactac
23281 tgatgctgtc cgtgatccac agacacttga gattcttgac attacaccat gttcttttgg
23341 tggtgtcagt gttataacac caggaacaaa tacttctaac caggttgctg ttctttatca
23401 ggatgttaac tgcacagaag tccctgttgc tattcatgca gatcaactta ctcctacttg
23461 gcgtgtttat tctacaggtt ctaatgtttt tcaaacacgt gcaggctgtt taataggggc
23521 tgaacatgtc aacaactcat atgagtgtga catacccatt ggtgcaggta tatgcgctag

23581 ttatcagact cagactaatt ctcctcggcg ggcacgtagt gtagctagtc aatccatcat
23641 tgcctacact atgtcacttg gtgcagaaaa ttcagttgct tactctaata actctattgc
23701 catacccaca aattttacta ttagtgttac cacagaaatt ctaccagtgt ctatgaccaa
23761 gacatcagta gattgtacaa tgtacatttg tggtgattca actgaatgca gcaatctttt
23821 gttgcaatat ggcagttttt gtacacaatt aaaccgtgct ttaactggaa tagctgttga
23881 acaagacaaa aacacccaag aagtttttgc acaagtcaaa caaatttaca aaacaccacc
23941 aattaaagat tttggtggtt ttaatttttc acaaatatta ccagatccat caaaaccaag
24001 caagaggtca tttattgaag atctactttt caacaaagtg acacttgcag atgctggctt
24061 catcaaacaa tatggtgatt gccttggtga tattgctgct agagacctca tttgtgcaca
24121 aaagtttaac ggccttactg ttttgccacc tttgctcaca gatgaaatga ttgctcaata
24181 cacttctgca ctgttagcgg gtacaatcac ttctggttgg acctttggtg caggtgctgc
24241 attacaaata ccatttgcta tgcaaatggc ttataggttt aatggtattg gagttacaca
24301 gaatgttctc tatgagaacc aaaaattgat tgccaaccaa tttaatagtg ctattggcaa
24361 aattcaagac tcactttctt ccacagcaag tgcacttgga aaacttcaag atgtggtcaa
24421 ccaaaatgca caagctttaa acacgcttgt taaacaactt agctccaatt ttggtgcaat
24481 ttcaagtgtt ttaaatgata tcctttcacg tcttgacaaa gttgaggctg aagtgcaaat
24541 tgataggttg atcacaggca gacttcaaag tttgcagaca tatgtgactc aacaattaat
24601 tagagctgca gaaatcagag cttctgctaa tcttgctgct actaaaatgt cagagtgtgt
24661 acttggacaa tcaaaaagag ttgatttttg tggaaagggc tatcatctta tgtccttccc
24721 tcagtcagca cctcatggtg tagtcttctt gcatgtgact tatgtccctg cacaagaaaa
24781 gaacttcaca actgctcctg ccatttgtca tgatggaaaa gcacactttc ctcgtgaagg
24841 tgtctttgtt tcaaatggca cacactggtt tgtaacacaa aggaattttt atgaaccaca
24901 aatcattact acagacaaca catttgtgtc tggtaactgt gatgttgtaa taggaattgt
24961 caacaacaca gtttatgatc ctttgcaacc tgaattagac tcattcaagg aggagttaga
25021 taaatatttt aagaatcata catcaccaga tgttgattta ggtgacatct ctggcattaa
25081 tgcttcagtt gtaaacattc aaaaagaaat tgaccgcctc aatgaggttg ccaagaattt
25141 aaatgaatct ctcatcgatc tccaagaact tggaaagtat gagcagtata taaaatggcc
25201 atggtacatt tggctaggtt ttatagctgg cttgattgcc atagtaatgg tgacaattat
25261 gctttgctgt atgaccagtt gctgtagttg tctcaagggc tgttgttctt gtggatcctg
25321 ctgcaaattt gatgaagacg actctgagcc agtgctcaaa ggagtcaaat tacattacac
25381 ataaacgaac ttatggattt gtttatgaga atcttcacaa ttggaactgt aactttgaag
25441 caaggtgaaa tcaaggatgc tactccttca gattttgttc gcgctactgc aacgataccg
25501 atacaagcct cactcccttt cggatggctt attgttggcg ttgcacttct tgctgttttt
25561 cagagcgctt ccaaaatcat aaccctcaaa aagagatggc aactagcact ctccaagggt
25621 gttcactttg tttgcaactt gctgttgttg tttgtaacag tttactcaca ccttttgctc
25681 gttgctgctg gccttgaagc cccttttctc tatctttatg ctttagtcta cttcttgcag
25741 agtataaact ttgtaagaat aataatgagg ctttggcttt gctggaaatg ccgttccaaa
25801 aacccattac tttatgatgc caactatttt ctttgctggc atactaattg ttacgactat
25861 tgtatacctt acaatagtgt aacttcttca attgtcatta cttcaggtga tggcacaaca
25921 agtcctattt ctgaacatga ctaccagatt ggtggttata ctgaaaaatg ggaatctgga

25981 gtaaaagact gtgttgtatt acacagttac ttcacttcag actattacca gctgtactca
26041 actcaattga gtacagacac tggtgttgaa catgttacct tcttcatcta caataaaatt
26101 gttgatgagc ctgaagaaca tgtccaaatt cacacaatcg acggttcatc cggagttgtt
26161 aatccagtaa tggaaccaat ttatgatgaa ccgacgacga ctactagcgt gcctttgtaa
26221 gcacaagctg atgagtacga acttatgtac tcattcgttt cggaagagac aggtacgtta
26281 atagttaata gcgtacttct ttttcttgct ttcgtggtat tcttgctagt tacactagcc
26341 atccttactg cgcttcgatt gtgtgcgtac tgctgcaata ttgttaacgt gagtcttgta
26401 aaaccttctt tttacgttta ctctcgtgtt aaaaatctga attcttctag agttcctgat
26461 cttctggtct aaacgaacta aatattatat tagtttttct gtttggaact ttaattttag
26521 ccatggcaga ttccaacggt actattaccg ttgaagagct taaaaagctc cttgaacaat
26581 ggaacctagt aataggtttc ctattcctta catggatttg tcttctacaa tttgcctatg
26641 ccaacaggaa taggtttttg tatataatta agttaatttt cctctggctg ttatggccag
26701 taactttagc ttgttttgtg cttgctgctg tttacagaat aaattggatc accggtggaa
26761 ttgctatcgc aatggcttgt cttgtaggct tgatgtggct cagctacttc attgcttctt
26821 tcagactgtt tgcgcgtacg cgttccatgt ggtcattcaa tccagaaact aacattcttc
26881 tcaacgtgcc actccatggc actattctga ccagaccgct tctagaaagt gaactcgtaa
26941 tcggagctgt gatccttcgt ggacatcttc gtattgctgg acaccatcta ggacgctgtg
27001 acatcaagga cctgcctaaa gaaatcactg ttgctacatc acgaacgctt tcttattaca
27061 aattgggagc ttcgcagcgt gtagcaggtg actcaggttt tgctgcatac agtcgctaca
27121 ggattggcaa ctataaatta aacacagacc attccagtag cagtgacaat attgctttgc
27181 ttgtacagta agtgacaaca gatgtttcat ctcgttgact ttcaggttac tatagcagag
27241 atattactaa ttattatgag gacttttaaa gtttccattt ggaatcttga ttacatcata
27301 aacctcataa ttaaaaattt atctaagtca ctaactgaga ataaatattc tcaattagat
27361 gaagagcaac caatggagat tgattaaacg aacatgaaaa ttattctttt cttggcactg
27421 ataacactcg ctacttgtga gctttatcac taccaagagt gtgttagagg tacaacagta
27481 cttttaaaag aaccttgctc ttctggaaca tacgagggca attcaccatt tcatcctcta
27541 gctgataaca aatttgcact gacttgcttt agcactcaat ttgcttttgc ttgtcctgac
27601 ggcgtaaaac acgtctatca gttacgtgcc agatcagttt cacctaaact gttcatcaga
27661 caagaggaag ttcaagaact ttactctcca atttttctta ttgttgcggc aatagtgttt
27721 ataacacttt gcttcacact caaaagaaag acagaatgat tgaactttca ttaattgact
27781 tctatttgtg ctttttagcc tttctgctat tccttgtttt aattatgctt attatctttt
27841 ggttctcact tgaactgcaa gatcataatg aaacttgtca cgcctaaacg aacatgaaat
27901 ttcttgtttt cttaggaatc atcacaactg tagctgcatt tcaccaagaa tgtagtttac
27961 agtcatgtac tcaacatcaa ccatatgtag ttgatgaccc gtgtcctatt cacttctatt
28021 ctaaatggta tattagagta ggagctagaa aatcagcacc tttaattgaa ttgtgcgtgg
28081 atgaggctgg ttctaaatca cccattcagt acatcgatat cggtaattat acagtttcct
28141 gttcaccttt tacaattaat tgccaggaac ctaaattggg tagtcttgta gtgcgttgtt
28201 cgttctatga agacttttta gagtatcatg acgttcgtgt tgttttagat ttcatctaaa
28261 cgaacaaact aaaatgtctg ataatggacc ccaaaatcag cgaaatgcac cccgcattac
28321 gtttggtgga ccctcagatt caactggcag taaccagaat ggagaacgca gtggggcgcg

28381 atcaaaacaa cgtcggcccc aaggtttacc caataatact gcgtcttggt tcaccgctct
28441 cactcaacat ggcaaggaag accttaaatt ccctcgagga caaggcgttc caattaacac
28501 caatagcagt ccagatgacc aaattggcta ctaccgaaga gctaccagac gaattcgtgg
28561 tggtgacggt aaaatgaaag atctcagtcc aagatggtat ttctactacc taggaactgg
28621 gccagaagct ggacttccct atggtgctaa caaagacggc atcatatggg ttgcaactga
28681 gggagccttg aatacaccaa aagatcacat tggcacccgc aatcctgcta acaatgctgc
28741 aatcgtgcta caacttcctc aaggaacaac attgccaaaa ggcttctacg cagaagggag
28801 cagaggcggc agtcaagcct cttctcgttc ctcatcacgt agtcgcaaca gttcaagaaa
28861 ttcaactcca ggcagcagta ggggaacttc tcctgctaga atggctggca atggcggtga
28921 tgctgctctt gctttgctgc tgcttgacag attgaaccag cttgagagca aaatgtctgg
28981 taaaggccaa caacaacaag gccaaactgt cactaagaaa tctgctgctg aggcttctaa
29041 gaagcctcgg caaaaacgta ctgccactaa agcatacaat gtaacacaag cttttggcag
29101 acgtggtcca gaacaaaccc aaggaaattt tggggaccag gaactaatca gacaaggaac
29161 tgattacaaa cattggccgc aaattgcaca atttgccccc agcgcttcag cgttcttcgg
29221 aatgtcgcgc attggcatgg aagtcacacc ttcgggaacg tggttgacct acacaggtgc
29281 catcaaattg gatgacaaag atccaaattt caaagatcaa gtcattttgc tgaataagca
29341 tattgacgca tacaaaacat tcccaccaac agagcctaaa aaggacaaaa agaagaaggc
29401 tgatgaaact caagccttac cgcagagaca gaagaaacag caaactgtga ctcttcttcc
29461 tgctgcagat ttggatgatt tctccaaaca attgcaacaa tccatgagca gtgctgactc
29521 aactcaggcc taaactcatg cagaccacac aaggcagatg ggctatataa acgttttcgc
29581 ttttccgttt acgatatata gtctactctt gtgcagaatg aattctcgta actacatagc
29641 acaagtagat gtagttaact ttaatctcac atagcaatct ttaatcagtg tgtaacatta
29701 gggatgactt gaaagagcca ccacattttc accgaggcca cgcggagtac gatcgagtgt
29761 acagtgaaca atgctaggga gagctgccta tatggaagag ccctaatgtg taaaattaat
29821 tttagtagtg ctatccccat gtgattttaa tagcttctta ggagaatgac aaaaaaaaaa
29881 a
